# The effect of non-analytical corrections on the phononic thermal transport in In*X* (*X* = S, Se, Te) monolayers

**DOI:** 10.1038/s41598-020-57644-0

**Published:** 2020-01-23

**Authors:** Aamir Shafique, Young-Han Shin

**Affiliations:** Department of Physics, University of Ulsan, Ulsan, 44610 Republic of Korea

**Keywords:** Thermoelectric devices and materials, Electronic properties and materials

## Abstract

We investigate the effect of non-analytical corrections on the phonon thermal transport properties in two-dimensional indium chalcogenide compounds. The longitudinal optical (LO) and transverse optical (TO) branches in the phonon dispersion are split near the Γ-point. The lattice thermal conductivity of monolayer InS is increased by 30.2% under non-analytical corrections because of the large LO-TO splitting at Γ-point. The predicted lattice thermal conductivities with non-analytical corrections at room temperature are 57.1 W/mK, 44.4 W/mK and 33.1 W/mK for the monolayer InS, InSe and InTe, respectively. The lattice thermal conductivity can be effectively reduced by nanostructures because the representative mean free paths are found very large in these monolayers. By quantifying the relative contribution of the phonon modes to the lattice thermal conductivity, we predict that the longitudinal acoustic branch is the main contributor to the lattice thermal conductivity. Due to the low lattice thermalconductivities of these monolayers, they can be useful in the nanoscale thermoelectric devices.

## Introduction

In the light of recent developments, a considerable amount of fundamental research and engineering applications are focused on the energy efficiency. One broad area of such scientific researches is based on the fundamental understanding of thermal transport processes of heat and how to employ it in our environment and materials^1–4^. Thermal transport research has promoted a diverse spectrum of the applications including high-performance thermoelectric materials to convert waste heat into useful electrical energy, thermal management in the nanoscale electronics, thermal barriers in modern construction, and the proposed use of nanoparticles in thermal medical therapies^5–8^. Therefore, understanding the thermal transport is very important for our basic knowledge of solid state physics and science.

Two-dimensional materials have been widely investigated in the past decade due to their extraordinary electrical, thermal, chemical and optical properties, and diverse spectrum of applications such as energy conversion, energy storage, nanoelectronics, and thermal management^9–12^. The thermal transport of two-dimensional materials is often essential in these applications, for example, low lattice thermal conductivity is required to convert waste energy into useful electricity and high lattice thermal conductivity is required for the thermal interface material. Many studies have been done based on first-principles calculations and classical molecular dynamics in search of the ideal two-dimensional thermoelectric material with a low lattice thermal conductivity^13–17^. Two-dimensional semiconductors such as silicene^18^, borophene^19^, stanene^20^, arsenene^21^, phosphorene^15^, and monolayers SnSe^16,22^, MoS_2_^23^, WSe_2_^24^ are extensively studied and explored for low lattice thermal conductivity, but efforts are still needed to find materials which show better electronic transport properties as well as low lattice thermal conductivity.

Two-dimensional indium chalcogenide compounds are polar materials due to the considerable charge transfer between indium and chalcogen atoms, which causes the creation of dipoles. A long-ranged electric field is generated by the the polarization density as a consequence of polarity in these materials, which produces the interaction between the dipoles. This dipole-dipole interaction strongly affects the frequencies of the optical branches and splits the longitudinal optical (LO) and transverse optical (TO) branches near the Γ-point. It has been shown both theoretically and experimentally that the long-range dipole-dipole interactions lead to LO-TO splitting near the Γ-point in bulk InSe^25,26^. We expect stronger dipole-dipole interaction in In*X* monolayers compared to their bulk counterparts because of smaller dielectric permittivity. Thus, the effect of dipole-dipole interaction on the phonon spectra and lattice thermal conductivity of In*X* monolayers would be much stronger than their bulk and meaningful. Secondly, we expect low lattice conductivities in these monolayers because of the low elastic moduli in comparison to other two-dimensional materials^27^ and these monolayers contain heavy elements such as In, Te, and Se. Wickramaratne *et al*. have predicted excellent electronic thermoelectric properties for these monolayers^28^, but there is a lack of study on phononic thermal transport properties which motivates us to study.

Here, we present a comprehensive study on the phonon transport properties in monolayer In*X* by solving the phonon Boltzmann transport equation (PBTE) based on first-principles calculations. The long-wavelength dispersion of longitudinal optical branches and lattice thermal conductivites of these monolayers are strongly affected by the non-analytical corrections to the dynamical matrix. The long-wavelength dispersion of longitudinal optical branches and lattice thermal conductivities of these monolayers are strongly affected by the non-analytical corrections to the dynamical matrix. The LO-TO splitting in monolayer InS is ten times larger than monolayer MoS_2_, which strongly affects the lattice thermal conductivity of monolayer InS. The lattice thermal conductivity trend (${\kappa }_{{\rm{InS}}} > {\kappa }_{{\rm{InSe}}} > {\kappa }_{{\rm{InTe}}}$) is explained with the help of the phonon spectra and their anharmonicities. Furthermore, the contribution of each mode toward total lattice thermal conductivity is extracted. We also discuss the dependence of the lattice thermal conductivity on temperature and size.

## Results and Discussions

**Figure 1:**
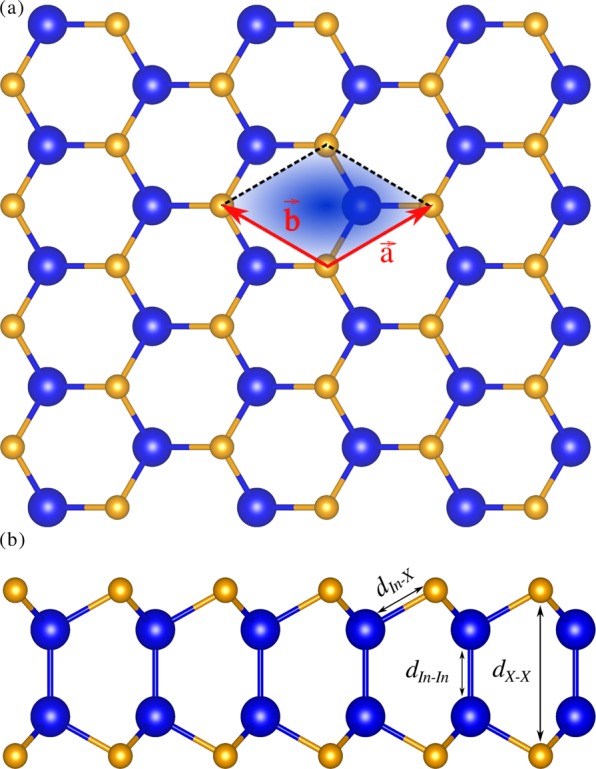
Atomic crystal structure of monolayer In*X* (*X*= S, Se, Te) from the (**a**) top and (**b**) side views. The arrows indicate the lattice vectors $\overrightarrow{a}$ and $\overrightarrow{b}$ and the dashed lines represent a primitive unit cell. Blue and brown spheres represent the In and *X* atoms, respectively.

Bulk indium chalcogenides exist in rhombohedral, tetragonal, cubic, orthorhombic, monoclinic, and hexagonal structures^29–31^. Here, we study only the energetically and dynamically stable hexagonal monolayer of indium chalcogenides with space group $P\bar{6}m2$ (187) with four atoms in a primitive unit cell as shown in Fig. 1. The optimized lattice parameters of monolayer InS, InSe, and InTe are *a* = *b* = 3.919 Å, 4.093 Å, and 4.382 Å, respectively, and they agree well with previous reports^32^ (See Table 1). The vertical distance between two chalcogen atoms (*d*_*X*−*X*_) is used as a thickness in the lattice thermal conductivity calculation.

**Table 1:** The calculated lattice constants (*a*), the vertical distance between Indium atoms (*d*_In−In_), the distance between Indium and chalcogen atoms (*d*_In−*X*_), and the vertical distance between chalcogen atoms of the monolayer In*X*.

	*a* (Å)	*d*_In−In_ (Å)	*d*_In−*X*_ (Å)	*d*_*X*−*X*_ (Å)
InS	3.919 (3.92)	2.827 (2.83)	2.551	5.182 (5.18)
InSe	4.093 (4.09)	2.816 (2.83)	2.689	5.385 (5.38)
InTe	4.382 (4.38)	2.823 (2.82)	2.884	5.596 (5.60)

The values in parentheses are taken from ref. ^32^.

**Figure 2:**
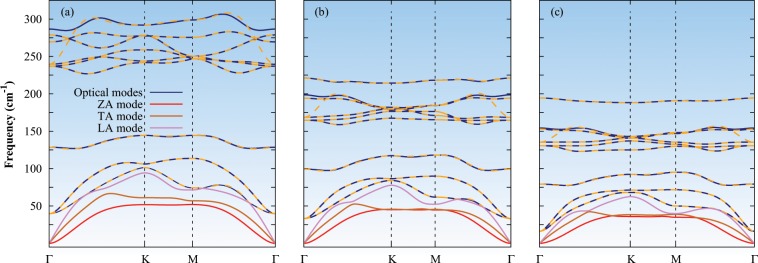
Phonon band structures of the monolayer (**a**) InS, (**b**) InSe, and (**c**) InTe along high-symmetry points Γ-K-M-Γ. Solid lines represent the phonon spectra in which non-analytical correction is included, and yellow dash lines represent the optical branches without non-analytical corrections.

The phonon band structures are shown in Fig. 2. Two in-plane acoustic modes (longitudinal acoustic (LA) and transverse acoustic (TA) modes) are linear near at the Γ point, and one out-of-plane acoustic mode (flexural acoustic (ZA) mode) has a quadratic nature near the Γ-point. The quadratic nature of ZA mode is a common feature of the two-dimensional materials, and it studied very well for graphene^13^, hexagonal boron nitride^33^, silicene^18^, and monolayer MoS_2_^34^. In two-dimension systems, the total energy of the system remain the same under a global rotation due to the invariant mechanics with respect to the orientation of their reference system. This enforces an additional linear constraints on usual acoustic sum rules of the force constants derived from translational symmetry which results the quadratic nature near the Γ-point. The ZA mode is critical in thermal transport because it contributes the major part of the lattice thermal conductivity in graphene^13^. The absence of the imaginary line in phonon band structures confirms the dynamical stability of these monolayers. The phonon dispersions of these monolayers look similar, and the band gap between low-frequency optical modes and high-frequency optical modes is 82.4 cm^−1^, 41.3 cm^−1^, and 27.8 cm^−1^ for the monolayer InS, InSe, and InTe, respectively.

The non-analytical corrections are applied to the dynamical matrix by calculating the dielectric constants and Born effective charges as summarized in Table 2. The corrections split longitudinal optical (LO) and transverse optical (TO) branches at the Γ-point in these monolayers as shown in Fig. 2. These LO-TO splitting are very strong and they are about ten times larger than monolayer MoS_2_. The polarization density produced by the atomic displacement (${u}_{LO}^{a}$) and the associated long-range electric fields are the responsible for the LO-TO splitting. The polarization density (*P*) Fourier transform can be written as^35^:

1 $$P({q}_{p})=\frac{{e}^{2}}{V}\sum _{a}\,{Z}_{a}\cdot {u}_{LO}^{a}$$

where *e* is the electron charge, *q*_*p*_ is the in-plane phonon momentum, and *Z*_*a*_ is the Born effective charge tensor associated with atom *a*. The polarization charge density (${q}_{p}P({q}_{p})$) is zero for the TO branch because the direction of propagation and the polarization is orthogonal to each other and the LO branch produces an electric field. The restoring force on the atoms is increased due to the electric field, and additional energy is required for the displacement of the LO branch with respect to the TO branch. The relationship between the frequency squares of these branches can be expressed as^35^:

2 $${\omega }_{LO}^{2}={\omega }_{TO}^{2}+{W}_{c}({q}_{p})\frac{{e}^{2}|{q}_{p}{|}^{2}}{V}{\left(\sum _{a}\frac{{e}_{{q}_{p}}\cdot {Z}_{a}\cdot {e}_{LO}^{a}}{\sqrt{{M}_{a}}}\right)}^{2}$$

where ${W}_{c}({q}_{p})=2\pi /|{q}_{p}|{\varepsilon }_{2D}(|{q}_{p}|)$ is the screened Coulomb interaction (which is inversely proportional to the dielectric constant) and ${e}_{{q}_{p}}={q}_{p}/|{q}_{p}|$ is momentum unit vector. The LO-TO splitting depends on the screened Coulomb interaction and the momentum direction (*q*_*p*_) along the Born effective charges. The large LO-TO splitting in the monolayer InS, compare to monolayer InSe and InTe because the dipole moment is strongly dependent on the electronegativity difference, the larger the difference in electronegativity results the larger dipole moment. When we go down the group (from S to Te), the electronegativity decreases which decreases the dipole moment and hence the Coluomb’s interaction. The second reason of the large LO-TO splitting in the monolayer is the lower mass of the S.

**Table 2:** The dielectric constants and Born effective charges of the monolayer In*X*.

	*ε*_*xx*_ = *ε*_*yy*_	*ε*_*zz*_	In		*X*	
			${{\boldsymbol{Z}}}_{{\boldsymbol{xx}}}^{\ast }={{\boldsymbol{Z}}}_{{\boldsymbol{yy}}}^{\ast }$ (*e*)	${{\boldsymbol{Z}}}_{{\boldsymbol{zz}}}^{\ast }$(*e*)	${{\boldsymbol{Z}}}_{{\boldsymbol{xx}}}^{\ast }={{\boldsymbol{Z}}}_{{\boldsymbol{yy}}}^{\ast }$ (*e*)	${{\boldsymbol{Z}}}_{{\boldsymbol{zz}}}^{\ast }$ (*e*)
InS	2.912	1.373	2.466	0.293	−2.466	−0.293
InSe	3.305	1.409	2.505	0.250	−2.505	−0.250
InTe	4.024	1.462	2.368	0.200	−2.368	−0.200

**Figure 3:**
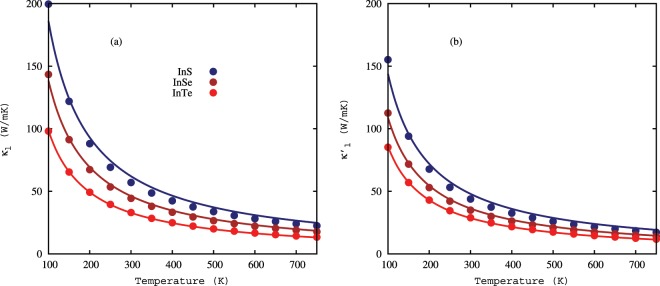
Lattice thermal conductivities of the monolayer InS, InSe, and InTe as a function of temperature calculated using iterative solutions of the phonon Boltzmann transport equation, (**a**) lattice thermal conductivity (${\kappa }_{l}$) with non-analytical corrections, and (**b**) lattice thermal conductivity (${\kappa ^{\prime} }_{l}$) without non-analytical corrections. Solid lines are from the fitting of lattice thermal conductivities to 1/*T*.

The lattice thermal conductivities as a function temperature are plotted for the InS, InSe, and InTe monolayers in Fig. 3(a). The lattice thermal conductivities decrease in the temperature range from 100 K to 750 K, and they are fitted well with the ${\kappa }_{l} \sim 1/T$ relationship, which demonstrates that the dominant three-phonon scattering processes in this temperature range are the Umklapp process. The lattice thermal conductivities of the three monolayers are 57.09 W/mK (InS), 44.43 W/mK (InSe), and 33.05 W/mK (InTe) at room temperature. Our calculated value of lattice thermal conductivity for the monolayer InSe agrees well with the recently reported value^36^. They possess low lattice thermal conductivity, especially for monolayer InTe. The lattice thermal conductivity of InTe is lower as compared to a lot of other two-dimensional materials, such as silicene^18^, phosphorene^15^, hexagonal boron nitride^37^, and monolayer MoS_2_^23^. The lattice thermal conductivities of the monolayers In*X* are higher than monolayer SnSe^22^, SnS^22^, SnSe_2_^38^, SnS_2_^38^, and stanene^20^. The possible reasons of lower lattice conductivity in monolayer InTe are the small phonon band gap between the optical modes because the small gap causes stronger scattering between the optical modes phonon and heavy mass of In and Te.

We have also computed the lattice thermal conductivities without the non-analytical corrections as shown in Fig. 3(b) to estimate the effect of LO-TO splitting and we found that the lattice thermal conductivities are strongly affected by the dipole-dipole interactions. The lattice thermal conductivities without the non-analytical corrections are decreased by 23.17%, 21.20%, and 12.62% for monolayer InS, InSe, and InTe, respectively. The non-analytical corrections will shift the optical bands in a neighborhood of Γ-point, thus changing the amount of phase space available for three-phonon scattering. The phase space for these monolayers increases substantially when we remove the non-analytical corrections, which explains the increase in thermal conductivities.

The lattice thermal conductivities are changed drastically in the low-temperature range (100 K ~ 300 K), and this change is partially attributed to the low Debye temperature (${\Theta }_{D}^{\alpha }$) of the acoustic phonon modes as given in Table 3. The Debye temperature corresponds to the temperature at which a phonon mode starts to be excited, and it is defined as ${\Theta }_{D}^{\alpha }=\frac{h{\omega }_{m}^{\alpha }}{{k}_{B}}$, where *α* shows ZA, TA, and LA modes, and ${\omega }_{m}^{\alpha }$ is the maximum frequency of the corresponding phonon mode. More phonon modes are activated in this temperature range, and the population of phonons is increased. Enhancement in the phonon population leads to an increase in phonon scattering rates and hence, the lattice thermal conductivity is dramatically decreased.

**Table 3:** Debye temperature (${\Theta }_{D}^{\alpha }$), representative mean free path (rMFP), specific heat (*C*_*v*_), room temperature lattice thermal conductivity with non-analytical corrections (${\kappa }_{l}$), and lattice thermal conductivity without non-analytical corrections (${\kappa ^{\prime} }_{l}$) of the In*X* monolayers.

	${{\boldsymbol{\Theta }}}_{{\boldsymbol{D}}}^{{\boldsymbol{ZA}}}$ (K)	${{\boldsymbol{\Theta }}}_{{\boldsymbol{D}}}^{{\boldsymbol{TA}}}$ (K)	${{\boldsymbol{\Theta }}}_{{\boldsymbol{D}}}^{{\boldsymbol{LA}}}$ (K)	rMFP (nm)	*C*_*v*_ (10_5_ J/Km^3^)	*κ*_*l*_ (W/mK)	${{\boldsymbol{\kappa }}{\boldsymbol{^{\prime} }}}_{{\boldsymbol{l}}}$ (W/mK)
InS	74.79	95.57	135.73	535.16	4.64	57.09	43.86
InSe	65.34	75.64	111.96	774.07	4.40	44.43	35.01
InTe	52.89	55.28	90.03	350.75	3.89	33.05	28.88

The contribution of the ZA, TA, LA, and optical branches to the lattice thermal conductivity at room temperature is calculated as given in Table 4. The main contributor to the lattice thermal conductivity is the LA branch because of the large LA branch phonon group velocity and long phonon lifetime. In the case of graphene, the main contributor is ZA branch where ZA contributes by 76%^14^. In these monolayers, the acoustic branches are granted by approximately 85%, and optical branches are contributed approximately 15%. Optical branches contribute more significant as compared to graphene, stanene and monolayer MoS_2_.

**Table 4:** Percentage contribution of the ZA, TA, LA, and optical phonon branches to lattice thermal conductivity at room temperature for the monolayer InS, InSe, and InTe.

	ZA (%)	TA (%)	LA (%)	Optical (%)
InS	27.18	19.97	39.35	13.48
InSe	22.66	23.09	43.34	10.91
InTe	17.14	18.35	48.33	16.18
graphene	76^14^	15^14^	8^14^	1^14^
stanene	13.5^20^	26.9^20^	57.5^20^	2.1^20^
SnS_2_	29.85^38^	32.32^38^	29.53^38^	8.30^38^
SnSe_2_	36.26^38^	25.56^38^	33.89^38^	4.29^38^

The phonon properties are investigated to understand the underlying phenomena of lower lattice thermal conductivity in these monolayers and the trend of lattice thermal conductivity (InS > InSe > InTe). The solution of the PBTE within single mode relaxation time approximation (SMRTA), the lattice thermal conductivity of a two-dimensional material can be written as ${\kappa }_{l}^{xx}=\frac{1}{2}\sum _{\alpha }\,{C}_{v,\alpha }{v}_{x}^{\alpha }.({v}_{x}^{\alpha }+{\Delta }_{x}^{\alpha }){\tau }_{\alpha }$, where *C*_*v*,*α*_ is the specific heat. The phonon heat capacities for the monolayers In*X* are calculated using the relation: ${C}_{v,\alpha }=\frac{{k}_{B}}{VN}\sum _{\alpha }\,{\left(\frac{\hslash {\omega }_{\alpha }}{{k}_{B}T}\right)}^{2}{n}_{\alpha }^{0}({n}_{\alpha }^{0}+1)$ and the values are given in Table 3. The specific heat of the monolayer InTe is lower than those of the monolayers InS and InSe. The lower specific heat of the monolayer InTe is due to low vibrational frequency, and it is partially responsible for lower lattice thermal conductivity of InTe among monolayers In*X*.

**Figure 4:**
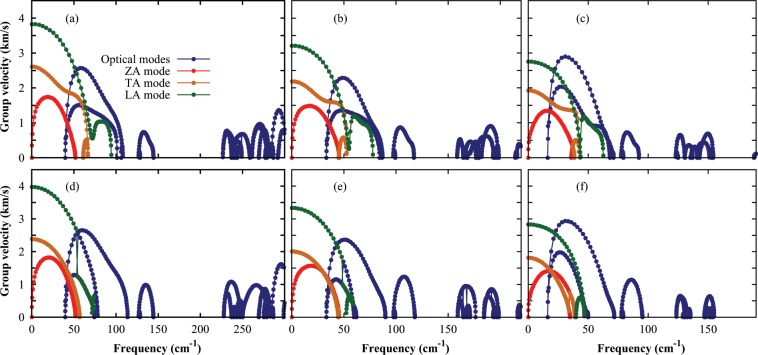
Branch-dependent phonon group velocities with non–analytical corrections in monolayer (**a**,**d**) InS, (**b**,**e**) InSe and (**c**,**f**) InTe along the Γ-K and Γ-M directions.

Phonon group velocity is an important factor that affects the lattice thermal conductivity. Phonon group velocities of monolayer InX are calculated along the Γ-M, and Γ-K directions as shown in Fig. 4 and they are determined from the slope of the phonon dispersion. Phonon group velocities of the monolayer InTe are found lower as compared to the monolayer InS and InSe and the acoustic phonon group velocities for monolayer InTe at the Γ-point are 1793 m/s and 2747 m/s for TA, and LA branches, respectively. The group velocities of the optical modes are very low as compared to the acoustic branches, and this low group velocities of the optical branches cause lower contribution to lattice thermal conductivity.

**Figure 5:**
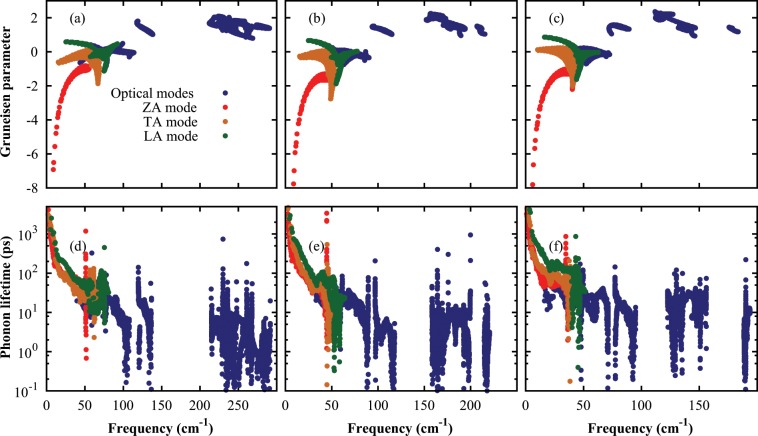
Mode dependent Grüneisen parameters and phonon lifetimes at room temperature as a function of frequency for the monolayer InS (**a**,**d**), InSe (**b**,**e**), and InTe (**c**,**f**), respectively.

The phonon lifetimes are extracted for each phonon mode in order to get more physical insight as shown in Fig. 5. The phonon-phonon scattering rates are dominated by isotopic and boundary scattering rates in the finite sample. In the monolayer InS, ZA mode is contributed 27.40% to the lattice thermal conductivity (larger than LA, TA and optical modes) because of longer phonon lifetime. However, LA mode is contributed more considerable in the monolayer InSe and InTe due to longer phonon lifetimes and large group velocity. The optical phonon lifetimes are very short that why they contribute very little to the lattice thermal conductivity.

Grüneisen parameter measures the anharmonicity in the chemical bonding, which drives the normal and umklapp phonon-phonon scattering processes. It is calculated from the change in phonon frequency with respect to change in lattice constant, and it can be expressed as:

$${\gamma }_{\alpha }=-\frac{{a}_{0}}{{\omega }_{\alpha }}\frac{\partial {\omega }_{\alpha }}{\partial a}$$

where *γ*_*α*_ is the Grüneisen parameter of the *α* branch and *a*_0_ is the equilibrium lattice constant. The *γ* for each phonon branch is computed as shown in Fig. 5(a–c) to clarify the origin of low lattice thermal conductivities in these monolayers. The *γ* for the ZA mode is inversely proportional to the wave vector squared (1/*q*^2^), It can be easily explained from the definition of Grüneisen parameter for two-dimensional system and from the quadratic phonon dispersion of the ZA mode. The *γ*_*ZA*_ is proportional to 1/*q*^2^ under small positive and negative strain, since the second term in *γ*_*ZA*_(*q*) will not depend on q. Similar behavior is found graphene and BN-dope graphene^39^. The *γ* are anomalously large for these monolayer In*X*, which lead to the low lattice thermal conductivity. Large Grüneisen parameters are the consequence of weak bonding in these monolayers. Strong anharmonicity leads to short phonon lifetime because phonon-phonon scattering rates also depend on anharmonicity of the material.

**Figure 6:**
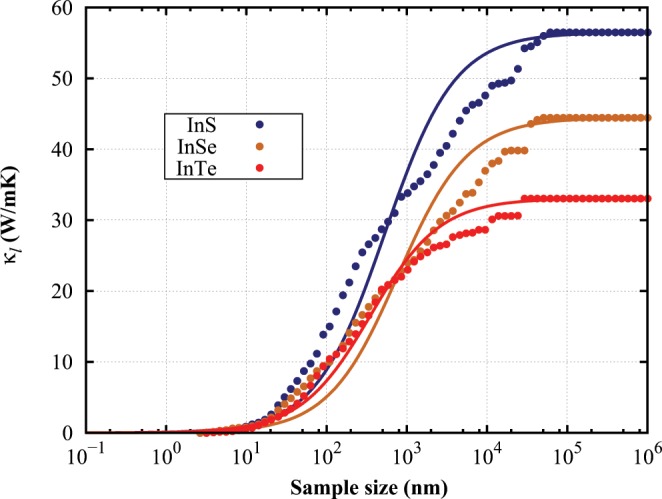
Cumulative lattice thermal conductivity with non-analytical corrections as a function of phonon mean free path for monolayer In*X* at room temperature. By fitting the ${\kappa }_{l}$ the Eq. 3, the fitted curves are plotted with solid lines.

The effect of size on the lattice thermal conductivity is significant in the nanoscale devices because when the sample size decreases from maximal phonon mean free path (MFP), the phonon-boundary scattering is increased and thus lattice thermal conductivity is decreased. To investigate the size-dependence, the cumulative lattice thermal conductivity as a function of phonon MFP is calculated as illustrated in Fig. 6 for the monolayer In*X*. The cumulative lattice thermal conductivity increases as phonon MFP increases and saturates at maximal phonon MFP. The maximal phonon MFP values for the monolayer InS, InSe and InTe, are 61.35 *μ*m, 35.11 *μ*m, and 29.15 *μ*m, respectively. The cumulative lattice thermal conductivity is fitted to a uniparametric function in Eq. 3 to evaluate the representative mean free path (rMPF, *L*_0_) and the fitted curves are shown in Fig. 6. The uniparametric function is given as^40^:

3 $${\kappa }_{l}(L)=\frac{{\kappa }_{l}^{max}}{1+\frac{{L}_{0}}{L}}$$

where ${\kappa }_{l}$ is the cumulative lattice thermal conductivity, and ${\kappa }_{l}^{max}$ is the maximal lattice thermal conductivity. The rMFP values are tabulated in Table 3, which are larger than those of phosphorene, monolayer SnS_2_ and SnSe_2_, and smaller than that of stanene^20,38^. The rMFP is very important in the designing of nanostructure because the phonon-boundary scattering dominates over the three-phonon scattering when the size of the sample below rMFP.

## Conclusions

In conclusions, phonon thermal transport properties, and temperature- and size-dependent lattice thermal conductivities of the monolayer In*X* investigated by employing first-principles calculations coupled with an iterative solution of the phonon Boltzmann transport equation The lattice thermal conductivity of these monolayers decreased with increasing temperature and perfectly follows the relation ${\kappa }_{l} \sim 1/T$. The predicted values of the lattice thermal conductivity at room temperature are low as compared to lots of other two-dimensional materials. The low lattice thermal conductivities originated from the strong anharmonicity, low phonon group velocity, low Debye temperature, and short phonon lifetimes. The lattice thermal conductivity can be effectively reduced by nanostructuring due to the large phonon MFP. Our work proposes that these materials can be considered for thermoelectric applications.

## Methods

When a material is placed in the temperature gradient (∇*T*), the phonon heat flux (*J*) is induced and it can be written as^41^:

4 $$J=\frac{1}{NV}\sum _{\alpha q}\,\hslash {\omega }_{\alpha q}{v}_{\alpha q}{n}_{\alpha q},$$

where *α* is the phonon branch index, *V* is the volume of the unit cell, *N* is the number of *q* points in the first Brillouin zone, and *ω*_*αq*_, *v*_*αq*_, and *n*_*αq*_ are the phonon frequency, the phonon group velocity, and the phonon distribution function, respectively. In the steady state, the phonon distribution function obeys the PBTE^41,42^:

5 $$-{\overrightarrow{v}}_{\alpha {\rm{q}}}\cdot \nabla T\left(\frac{\partial {n}_{\alpha q}}{\partial T}\right)+{\left(\frac{\partial {n}_{\alpha q}}{\partial t}\right)}_{scatt}=0.$$

The first term denotes the diffusion of the *n*_*αq*_ due to the temperature gradient and the second term depends on the different scattering processes, such as the phonon-phonon scattering, the phonon-boundary scattering, and the phonon-isotope scattering. For a small temperature gradient, *n*_*αq*_ changes from its equilibrium state: ${n}_{\alpha q}={n}_{\alpha q}^{0}+{n}_{\alpha q}^{1}$, where ${n}_{\alpha q}^{0}$ is the Bose-Einstein distribution function and ${n}_{\alpha q}^{1}$ is the fluctuation in the distribution function. For small temperature gradient, the *n*_*αq*_ can be determined by linearization of the Eq. 5^41^:

6 $${n}_{\alpha q}={n}_{\alpha q}^{0}-{\overrightarrow{F}}_{\alpha q}\cdot \nabla T\frac{\partial {n}_{\alpha q}^{0}}{\partial T}.$$

The term *F*_*αq*_ is expressed as^40^:

7 $${\overrightarrow{F}}_{\alpha q}={\tau }_{\alpha q}({\overrightarrow{v}}_{\alpha q}+{\overrightarrow{\Delta }}_{\alpha q})$$

where *τ*_*αq*_ is the phonon lifetime and ${\overrightarrow{\Delta }}_{\alpha q}$ is the correction term for the iterative solution of the PBTE. The correction term ${\overrightarrow{\Delta }}_{\alpha q}$ is zero in the case of the single-mode relaxation time approximation (SMRTA). According to Fourier’s law, the heat flux is directly proportional to the heat flux

8 $$\overrightarrow{J}=-\,{\kappa }_{l}\nabla T$$

where ${\kappa }_{l}$ is the lattice thermal conductivity tensor. It can be estimated by solving the Boltzmann transport equation and comparing Eqs. 8 to 4 as^40^:

9 $${\kappa }_{l}^{ij}=\frac{1}{{k}_{B}{T}^{2}NV}\sum _{\alpha q}\,{n}_{\alpha q}^{0}({n}_{\alpha q}^{0}+1){(\hslash {\omega }_{\alpha q})}^{2}{v}_{\alpha q}^{i}{F}_{\alpha q}^{j}$$

where *k*_*B*_ is the Boltzmann constant, *i* and *j* represent the Cartesian coordinates. The ${v}_{\alpha }^{i}$ is the phonon group velocity of the *α* branch along the direction *i*.

The phonon lifetime is calculated using the Matthiessen’s rule, which is given as:

10 $$\frac{1}{{\tau }_{\alpha q}}=\frac{1}{{\tau }_{\alpha q}^{3ph}}+\frac{1}{{\tau }_{\alpha q}^{iso}}+\frac{1}{{\tau }_{\alpha q}^{b}}$$

where $1/{\tau }_{\alpha q}^{3ph}$ is the three-phonon scattering rate, $1/{\tau }_{\alpha q}^{iso}$ is the phonon-isotope scattering rate, and $1/{\tau }_{\alpha q}^{b}$ is the phonon-boundary scattering rate. The three-phonon scattering rate is calculated as^40^:

11 $$\frac{1}{\tau_{\alpha q}^{3ph}}={\frac{1}{N}}\left(\sum_{{\alpha \prime}\ q{\prime}\ \alpha{\prime\prime}\ q{\prime \prime}} {\Gamma_{{\alpha q \alpha \prime}\ q{\prime}\ \alpha{\prime\prime}\ q{\prime \prime}}^{+}}+{\frac{1}{2}} \sum_{{\alpha \prime}\ q{\prime}\ \alpha{\prime\prime}\ q{\prime \prime}} {\Gamma_{{\alpha \prime}\ q{\prime}\ \alpha{\prime\prime}\ q{\prime \prime}}^{-}}\right)$$

where *α*′ and *α*″ represent the second and third phonon branch scattering with the phonon branch *α*, ${\Gamma }_{\alpha q\alpha ^{\prime} q^{\prime} \alpha ^{\prime\prime} q^{\prime\prime} }^{+}$ and ${\Gamma }_{\alpha q\alpha ^{\prime} q^{\prime} \alpha ^{\prime\prime} q^{\prime\prime} }^{-}$ are the three-phonon scattering rates of the absorption process (*α* + *α*′ → *α*″ and the emission process (*α* → *α*′ + *α*″), respectively. The phonon-isotope scattering rate can be obtained using the Tamura’s formula^43^

12 $$\frac{1}{{\tau }_{\alpha q}^{iso}}=\frac{\pi {\omega }^{2}}{2}\sum _{i}\,g(x){|{e}_{\alpha q}^{\ast }(x){e}_{\alpha ^{\prime} q^{\prime} }(x)|}^{2}\delta ({\omega }_{\alpha q}-{\omega }_{\alpha ^{\prime} q^{\prime} })$$

where $g(x)=\sum _{s}\,{f}_{s}(x){[1-{m}_{s}(x)/\bar{m}(x)]}^{2}$ denotes the Pearson deviation coefficient of the masses *m*_*s*_(*x*) of isotopes *s* of atoms *x*. The phonon-boundary scattering rate can be computed by the standard equation^44^

13 $$\frac{1}{{\tau }_{\alpha q}^{b}}=\frac{1-p}{1+p}\frac{|{v}_{\alpha q}|}{L},$$

where *p* is the specularity parameter which describes the roughness of the boundary and *L* is the system size.

The optimized lattice parameters and interatomic force constants (IFCs) are obtained from the total energy calculations by using plane augmented wave method^45^ based on density functional theory with Vienna ab initio simulation package (VASP)^46^. We use generalized gradient approximation (GGA) parameterized by the Perdew-Burke-Ernzerhof functional^47^ as exchange-correlation potential with a plane wave energy cutoff of 500 eV. All atoms in the unit cell are allowed to relax until the maximum force on each atom is smaller than 10^−4^ eV/Å with a *k*-point mesh of 25 × 25 × 1. A vacuum thickness of 25 Å is used in order to avoid interactions between the periodic images.

Harmonic force constants are determined using the finite displacement method as implemented in the Phonopy package^48^. A 6 × 6 × 1 supercell is used for the calculations of the phonon spectra, phonon group velocity, and harmonic force constants. A 5 × 5 × 1 supercell is employed for the anharmonic force constants including the fifth nearest-neighbor interaction. ShengBTE code^40^ is used to calculate the lattice thermal conductivity with a *q*-point mesh of 120 × 120 × 1.
